# Association between surgical volumes and hospital mortality in patients: a living donor liver transplantation single center experience

**DOI:** 10.1186/s12876-021-01732-6

**Published:** 2021-05-20

**Authors:** Chia-En Hsieh, Ya-Lan Hsu, Kuo-Hua Lin, Ping-Yi Lin, Yu-Ju Hung, Yi-Chun Lai, Li-Chueh Weng, Yao-Li Chen

**Affiliations:** 1 Department of Nursing, Changhua Christian Hospital, Changhua, Taiwan; 2 Graduate Institute of Clinical Medical Science, Chang Gung University, Taoyuan, Taiwan; 3 Department of General Surgery, Changhua Christian Hospital, No. 135 Nan-Hsiao Street, Changhua, 500 Taiwan; 4 Department of Nursing, Associate Professor, HungKung University, Taichung, Taiwan; 5 Department of Nursing, Graduate Institute of Clinical Medical Science, Chang Gung University, Taoyuan, 33302 Taiwan

**Keywords:** Living donor liver transplantation, Hospital mortality, Surgical volumes, Complication

## Abstract

**Background:**

Many factors cause hospital mortality (HM) after liver transplantation (LT).

**Methods:**

We performed a retrospective research in a single center from October 2005 to June 2019. The study included 463 living donor LT patients. They were divided into a no-HM group (n = 433, 93.52%) and an HM group (n = 30, 6.48%). We used logistic regression analysis to determine how clinical features and surgical volume affected HM. We regrouped patients based on periods of surgical volume and analyzed the clinical features.

**Results:**

Multivariate analysis revealed that donor age (OR = 1.050, 95% CI 1.011–1.091, *p* = 0.012), blood loss (OR = 1.000, 95% CI 1.000–1.000, *p* = 0.004), and annual surgical volumes being < 30 LTs (OR = 2.540, 95% CI 1.011–6.381, *p* = 0.047) were significant risk factors. A comparison of years based on surgical volume found that when the annual surgical volumes were at least 30 the recipient age (*p* = 0.023), donor age (*p* = 0.026), and ABO-incompatible operations (*p* < 0.001) were significantly higher and blood loss (*p* < 0.001), operative time (*p* < 0.001), intensive care unit days (*p* < 0.001), length of stay (*p* = 0.011), rate of re-operation (*p* < 0.001), and HM (*p* = 0.030) were significantly lower compared to when the annual surgical volumes were less than 30.

**Conclusions:**

Donor age, blood loss and an annual surgical volume < 30 LTs were significant pre- and peri-operative risk factors. Hospital mortality and annual surgical volume were associated with statistically significant differences; surgical volume may impact quality of care and transplant outcomes.

## Background

Liver transplantation (LT) is a major and difficult abdominal operation, and it involves multiple teams administering such things as anesthesia, color Doppler techniques, and critical care. Living donor liver transplantation (LDLT) was associated with a high rate of surgical complications after transplantation, and the hospital mortality rate after LDLT has ranged from 3.6 to 18.9% [1–3]. Factors related to in-hospital death include infection, a high model for end-stage liver disease (MELD) score, the recipient being of advanced age, and vascular complications such as hepatic artery thrombosis and portal vein thrombosis [3, 4]. Liver transplantation patients commonly acquire nosocomial infections, which can cause morbidity and mortality [5–7]. High MELD scores, large volume of blood loss, post-transplant hemodialysis, ABO incompatibility, and older donor age were independent risk factors for postoperative bacteremia [8–10]. In addition, high MELD scores, restrictive lung patterns and surgical complexity were risk factors with major impacts [11, 12].

However, centers with higher surgical volumes (based on annual liver transplantations) had better techniques and multiple team organization compared to centers with lower surgery volumes; higher in-hospital mortality was associated with lower surgical volume centers [1, 13]. In this paper, we therefore mainly analyzed hospital mortality in living donor liver transplant patients and tried to pinpoint the factors that influence postoperative prognosis in order to provide a reference for liver transplant teams.

## Methods

We performed a retrospective research in Changhua Christian Hospital from October 2005 to June 2019. Living liver grafts were from patients’ families, and none of the donors were prisoners who were executed. The study was approved by the Institutional Review Board of Changhua Christian Hospital (CCH 191244). The donors were selected based on general physical condition, blood tests, liver volumetry measured by a computed tomography scan, clinical psychological evaluation, and social assessment. A total of 463 living donor liver transplant patients were included in this study; none of them had combined liver and kidney transplantations. Four of the LTs involved left lobe grafts, and the other 459 involved right lobe grafts. The patients were divided into two groups: the no hospital mortality group (n = 433, 93.52%) and the hospital mortality group (n = 30, 6.48%).

## Definitions

**Fig. 1:**
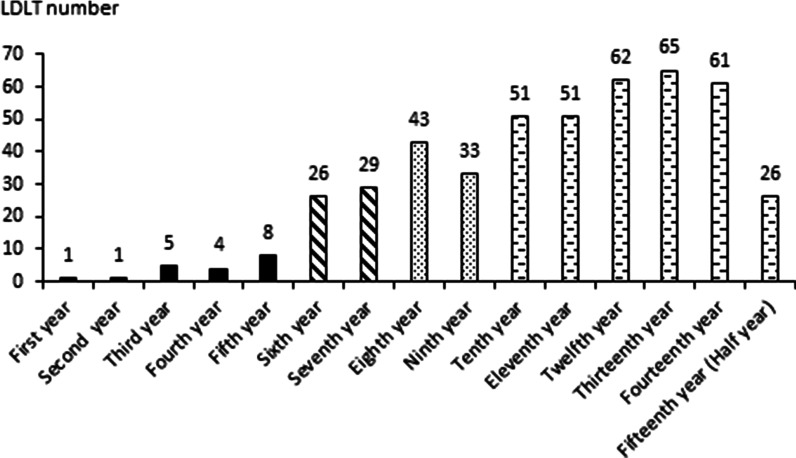
Living donor liver transplantations in our center. Annual LDLT volume was less than 10 cases in the first 5 years of our transplantation center (total 19 transplantation cases). It grew to 20–30 cases in the 6th and 7th years, over 30 cases after 8 years, and over 50 cases after 10 years

Renal failure (acute or chronic) was defined as the presence of a median glomerular filtration rate < 30 mL/min/1.73 m^2^ for at least 3–6 months or the need for long-term dialysis [14]. Biliary complication was defined as the presence of bile leakage or biliary stenosis. Re-operation was defined as the presence of a hemorrhage, vascular thrombosis (portal vein or hepatic artery thrombosis), abdomen abscess, or biliary complication that required another abdominal surgery. Hepatic artery complication was defined as the presence of thrombosis, dissection, stenosis, or steal blood flow with the need for thrombectomy, percutaneous transluminal angioplasty with stenting, or transarterial embolization for steal blood flow from the splenic artery. Portal vein complication was defined as the presence of thrombosis and stenosis with the need for thrombectomy and percutaneous transluminal angioplasty with stenting. Early allograft dysfunction was defined as the presence of at least 1 of the following parameters 7 days after liver transplantation: a serum bilirubin level ≥ 10 mg/dL, an international normalized ratio (INR) ≥ 1.6 or an alanine or aspartate aminotransferases (ALT or AST) level > 2000 IU/L [15]. Cardiovascular complication was defined as the presence of acute coronary syndrome, acute myocardial infarction or ruptured aortic mycotic aneurysms. Cerebrovascular complication was defined as the presence of subarachnoid hemorrhage, intracerebral hemorrhage, or central pontine myelinolysis. Pulmonary complication was defined as the presence of a lower respiratory tract infection, pneumonia or adult respiratory distress syndrome. Infection complication was defined as the presence of a bloodstream infection, intra-abdominal infection, or urinary tract infection. We distinguished pairs of surgical volume periods based on cutoffs of 20, 30, and 50 yearly liver transplantations (Fig. 1).

### Antimicrobial prophylaxis

Prophylaxis was administered intravenously from the day of transplantation. Piperacillin/tazobactam (Tazocin) as well as selective bowel decontamination (neomycin and nystatin administered orally) were used. Routine prophylactic antiviral therapy was not performed.

### Immunosuppression

All patients received calcineurin-inhibitor based initial immunosuppression. The majority also received and were maintained on cyclosporine or tacrolimus in combination with mycophenolate and methylprednisolone. The target levels after the first post-transplant year were as follows: 70–150 ng/mL for cyclosporine and 5–10 ng/mL for tacrolimus. Methylprednisolone was administered intravenously in four divided doses daily; the dosage was tapered from 200 to 20 mg/day over 6 days.

We had a program for ABO‐incompatible patients. First, we administered a preoperative anti‐CD20 antibody (rituximab, 375 mg/m^2^) treatment with preoperative plasma exchange to lower the anti‐AB antigen titer (1:32); second, we administered a postoperative anti‐CD20 antibody (rituximab, 187.5 mg/m^2^) treatment on post-liver transplantation day 1. Mycophenolate mofetil was given in doses of 0.5–1.5 g/day, and tacrolimus was kept at a trough level of 7–10 ng/dL. When the isoagglutinin titer was above 64, a plasma exchange was performed to lower the isoagglutinin titer to less than or equal to 64.

### Statistical analysis

The pre- and peri-operative periods had correlated clinical factors, including age, sex, MELD score, total bilirubin, creatinine, prothrombin time, INR, graft-recipient weight ratio (GRWR), blood loss, operative time, renal failure (acute or chronic), pre-LT in the intensive care unit, ABO-incompatible liver transplantation and surgical volume. All data were recorded on a computerized database. The patients were classified into two subgroups based on their hospital mortality status, and there were distinguished risk factors of liver transplantation. We regrouped patients based on surgical volume periods and analyzed the demographic data and clinical features. There were comparisons of pre-, peri-, and post-operative characteristics between the high and low surgical volume periods. The clinical post-operative factors included blood loss, operative time, intensive care unit days, length of stay, dialysis, re-operation, biliary complication, hepatic artery complication, portal vein complication, early allograft dysfunction, cardiovascular complication, cerebrovascular complication, pulmonary complication, hemorrhage and infection. Pearson’s chi-squared test and the independent *t* test were used to examine the differences between the two subgroups in terms of demographic factors and the clinical characteristics of the LDLT patients. Values for the continuous variables are presented as mean ± standard deviation (SD) in this study. Categorical variables were compared using the chi-squared test or Fisher exact test where appropriate. We used logistic regression analysis to determine how clinical features and surgical volume affected hospital mortality in liver transplantation patients. A *p* value less than 0.05 was considered to be statistically significant. The statistical analysis was performed with SPSS (Statistical Package for Social Science, version 20.0).

## Results

A total of 463 liver transplant patients were included in this study; they had a hospital mortality rate of 6.88%. The patients were divided into two groups: the no hospital mortality group (n = 433, 93.52%) and the hospital mortality group (n = 30, 6.48%). A comparison between the two groups found statistically significant differences in terms of senior donor age (*p* = 0.017), high MELD score (*p* < 0.001), blood loss (*p* = 0.014), annual surgical volume < 30 liver transplantations (*p* = 0.030), and pre-LT in the intensive care unit (< 0.001) (Table 1). For annual surgical volumes of more than 20, 30, and 50 living donor liver transplantations, the hospital mortality rates were 6.3%, 5.4%, and 5.1%, respectively. The 30 surgeries per year cutoff yielded statistically significant differences, unlike the other two cutoffs.

**Table 1:** Comparisons of demographic data and pre- and peri-operative clinical features based on hospital mortality in liver transplantation patients

Demographic and clinical features	No hospital mortality (n = 433)	Hospital mortality (n = 30)	*p*
	Mean ± SD (range)	Mean ± SD (range)	
Recipient age (years)	54.08 ± 8.41 (11–73)	52.43 ± 11.66 (21–73)	0.453
Donor age (years)	31.12 ± 9.15 (18.0–65.00)	35.27 ± 9.28 (20.0–56.00)	0.017
MELD score	17.59 ± 9.31 (3.0–40.0)	23.33 ± 9.32 (9.0–37.0)	< 0.001
Total bilirubin (mg/dL)	6.89 ± 9.62 (0.19–47.69)	8.75 ± 10.33 (0.32–38.68)	0.314
Creatinine (mg/dL)	1.16 ± 0.96 (0.32–8.65)	1.55 ± 1.10 (0.40–4.90)	0.075
INR	1.53 ± 0.638 (0.87–4.76)	1.69 ± 0.62 (0.95–3.64)	0.201
GRWR	1.10 ± 0.30 (0.55–2.28)	1.13 ± 0.33 (0.68–2.04)	0.617
Blood loss (ml)	3478.10 ± 3656.62 (300.0–39,450.0)	7003.00 ± 7371.99 (200.0–40,000.0)	0.014
Operative time (min)	408.79 ± 93.87 (215.00–855.00)	434.33 ± 105.65 (280.00–660.00)	0.154

	(%)	(%)	
Gender			0.859
Male	338 (78.1)	23 (76.7)	
Female	95 (21.9)	7 (23.3)	
The type of disease			0.087
Alcoholic ± viruses	108(24.9)	13(43.3)	
Virus related	284(65.6)	13(43.3)	
Primary biliary cholangitis	8(1.8)	1(3.3)	
Other	33(7.6)	3(10.0)	
Pre-LT renal failure			0.197
Yes	21 (4.8)	3 (10.0)	
No	412 (93.8)	27 (90.0)	
Pre-LT in intensive care unit			0.001
Yes	27 (6.2)	7 (23.3)	
No	406 (93.8)	23 (76.7)	
ABO incompatible	1.000		
Yes	51 (11.8)	3 (10.0)	
No	382 (88.2)	27 (90.0)	
Liver transplant cases/year			
< 20/year	17 (3.9)	2 (6.7)	0.353
≥ 20/year	416 (96.1)	28 (93.3)	
< 30/year	65 (15.0)	9 (30.0)	0.030
≥ 30/year	368 (85.0)	21 (70.0)	
< 50/year	135 (31.2)	14 (46.7)	0.079
≥ 50/year	298 (68.8)	16 (53.3)	

Model for end-stage liver disease (MELD) score, international normalized ratio (INR), graft-recipient weight ratio (GRWR), liver transplantation (LT). The types of disease were alcoholic (n = 102, 22.0%), alcoholic ± hepatitis B or C (n = 19, 4.1%), hepatitis B (n = 165, 35.6%), hepatitis C (n = 110, 23.8%), hepatitis B and C (n = 22, 4.8%), primary biliary cholangitis (n = 9, 1.9%) and other (n = 36, 7.8%): non-hepatitis B and C (n = 25), autoimmune (n = 6), drug toxicity (n = 1), liver tumor (n = 4)

Multivariate analysis revealed that donor age (OR = 1.050, 95% CI 1.011–1.091, *p* = 0.012), blood loss (OR = 1.000, 95% CI 1.000–1.000, *p* = 0.004) and annual surgical volume < 30 liver transplantations (OR = 2.540, 95% CI 1.011–6.381, *p* = 0.047) were significant risk factors from the pre- and peri-operative periods (Table 2). The high surgical volume period (annual surgical volume ≥ 30 liver transplantations) had significantly higher recipient age (*p* = 0.023), donor age (*p* = 0.026), and ABO-incompatible rate (*p* < 0.001) and significantly lower blood loss (*p* < 0.001), operative time (*p* < 0.001), intensive care unit days (*p* < 0.001), length of stay (*p* = 0.011), re-operation rate (*p* < 0.001), and hospital mortality rate (*p* = 0.030) compared to the low surgical volume period (annual surgical volume < 30 liver transplantations) (Table 3).

**Table 2:** Logistic regression analysis of factors affecting clinical features of hospital mortality in liver transplantation patients

Variable	Univariate analysis			Multivariate analysis		
	OR	95% CI	*p*	OR	95% CI	*p*
Recipient age (years)	0.979	0.941–1.020	0.313	–	–	–
Donor age (years)	1.044	1.007–1.083	0.019	1.050	1.011–1.091	0.012
MELD score	1.065	1.026–1.104	0.001	1.041	0.994–1.091	0.090
Total bilirubin (mg/dL)	1.019	0.985–1.054	0.278	–	–	–
Creatinine (mg/dL)	1.292	0.999–1.692	0.051	–	–	–
INR	1.370	0.842–2.229	0.205	–	–	–
GRWR	1.355	0.413–4.492	0.617	–	–	–
Blood loss (ml)	1.000	1.000–1.000	< 0.001	1.000	1.000–1.000	0.004
Operative time (min)	1.003	0.999–1.006	0.155	–	–	–
< 20 LT cases/year	1.748	0.384–7.946	0.470	–	–	–
< 30 LT cases/year	2.426	1.064–5.532	0.035	2.540	1.011–6.381	0.047
< 50 LT cases/year	1.931	0.916–4.071	0.084	–	–	–
Male	0.859	0.385–2.218	0.859	–	–	–
Pre-LT renal failure	2.180	0.612–7.769	0.229	–	–	–
Pre-LT in ICU	4.576	1.803–11.616	0.001	2.381	0.723–7.845	0.154
ABO incompatible	0.832	0.244–2.842	0.769	–	–	–

Model for end-stage liver disease (MELD) score, international normalized ratio (INR), graft-recipient weight ratio (GRWR), liver transplantation (LT), intensive care unit (ICU)

**Table 3:** Comparisons of demographic data and clinical features of surgical volume periods in liver transplantation patients

Demographic and clinical features	Annual surgical volumes < 30 (n = 74)	Annual surgical volumes ≥ 30 (n = 389)	*p*
	Mean ± SD (range)	Mean ± SD (range)	
Recipient age (years)	51.88 ± 9.33 (11–70)	54.37 ± 8.48 (18–73)	0.023
Donor age (years)	29.22 ± 8.13 (18.0–54.00)	31.80 ± 9.34 (18.0–65.00)	0.026
MELD score	17.32 ± 8.95 (6.0–40.0)	17.68 ± 9.43 (3.0–40.0)	0.661
Total bilirubin (mg/dL)	6.01 ± 9.10 (0.19–47.69)	7.01 ± 9.72 (0.32–37.94)	0.387
Creatinine (mg/dL)	1.13 ± 0.81 (0.38–4.90)	1.20 ± 0.99 (0.32–8.65)	0.764
INR	1.57 ± 0.71 (0.92–4.59)	1.54 ± 0.62 (0.87–4.76)	0.678
GRWR	1.15 ± 0.31 (0.65–2.16)	1.08 ± 0.30 (0.55–2.28)	0.105
Blood loss (ml)	6143.92 ± 5202.98 (200.0–23,000.0)	3242.82 ± 3661.38 (200.0–40,000.0)	< 0.001
Operative time (min)	474.51 ± 91.78 (290.00–730.00)	398.26 ± 90.24 (215.00–885.00)	< 0.001
APACHE II score	17.55 ± 7.00 (5.00–29.00)	18.46 ± 7.24 (3.00–37.00)	0.318
Intensive care unit (days)	15.23 ± 10.03(4.0–58.00)	9.63 ± 9.64 (1.00–114.00)	< 0.001
Length of stay (days)	35.55 ± 17.38 (14.00–119.00)	29.89 ± 17.38 (3.00–159.00)	0.011

	(%)	(%)	
Gender			0.081
Male	52 (70.3)	309 (79.4)	
Female	22 (29.7)	80 (20.6)	
Pre-LT renal failure			0.633
Yes	3 (4.1)	21 (5.4)	
No	71 (95.9)	368 (94.6)	
Pre-LT in intensive care unit			0.237
Yes	3 (4.1)	31 (8.0)	
No	71 (95.9)	358 (92.0)	
ABO incompatible			< 0.001
Yes	0 (0.0)	54 (13.9)	
No	74 (100.0)	335 (86.1)	
Dialysis			0.060
Yes	13 (17.6)	39 (10.0)	
No	61 (82.4)	350 (90.0)	
Re-operation			< 0.001
Yes	29 (39.2)	71 (18.3)	
No	45 (60.8)	318 (81.7)	
Biliary complication			0.160
Yes	12 (16.2)	41 (10.5)	
No	62 (83.8)	348 (89.5)	
Hepatic artery complication			0.849
Yes	3 (4.1)	14 (3.6)	
No	71 (95.9)	375 (96.4)	
Portal vein complication			0.366
Yes	0 (0.0)	8(2.1)	
No	74 (100.0)	381 (97.9)	
Early allograft dysfunction			0.376
Yes	0 (0.0)	10 (2.6)	
No	74 (100.0)	379 (97.4)	
Cardiovascular complication			0.161
Yes	3 (4.1)	6 (1.5)	
No	71 (95.9)	383 (98.5)	
Cerebrovascular complication			1.000
Yes	1 (1.4)	6 (1.5)	
No	73 (98.6)	383 (98.5)	
Pulmonary complication			0.183
Yes	12 (16.2)	42 (10.8)	
No	62 (83.8)	347 (89.2)	
Hemorrhage			0.099
Yes	13 (17.6)	42 (10.8)	
No	61 (82.4)	347 (89.2)	
Infection			0.187
Yes	28 (37.8)	117 (30.1)	
No	46 (62.2)	272 (69.9)	
Hospital mortality			0.030
Yes	9 (12.2)	21 (5.4)	
No	65 (87.8)	368 (94.6)	

Model for end-stage liver disease (MELD) score, international normalized ratio (INR), graft-recipient weight ratio (GRWR), liver transplantation (LT)

The most common cause of hospital death was infection (n = 13, 43.3%), and other causes often coincided with infection: hemorrhage with/without infection (n = 4, 13.3%), cerebrovascular with/without infection (n = 3, 10.0%), cardiovascular with/without infection (n = 6, 20.0%), and early allograft dysfunction with infection (n = 4, 13.3%). Most causes of patient death were major post-LT complications, which developed into septic shock and led to mortality. In the hospital mortality group, the high surgical volume period (annual surgical volume ≥ 30 liver transplantations) had significantly lower rates of hemorrhage (*p* ≤ 0.001), cerebrovascular complication (*p* < 0.001), and cardiovascular complication (*p* < 0.001) and a significantly higher rate of early allograft dysfunction (*p* < 0.001) compared to the low surgical volume period (annual surgical volume < 30 liver transplantations) by nonparametric statistics. (Table 4).

**Table 4:** Comparisons of causes of mortality and surgical volume periods in hospital mortality of liver transplantation patients

	Annual surgical volumes < 30 n = 9 (%)	Annual surgical volumes ≥ 30 n = 21 (%)	*p*
Infection			0.465
Yes	4 (44.4)	9 (42.9)	
No	5 (55.6)	12 (57.1)	
Hemorrhage with/without infection			< 0.001
Yes	2 (22.2)	2 (9.5)	
No	7 (77.8)	19 (90.5)	
Cerebrovascular with/without infection			< 0.001
Yes	1 (11.1)	2 (9.5)	
No	8 (88.9)	19 (90.5)	
Cardiovascular with/without infection			0.001
Yes	2 (22.2)	4 (19.0)	
No	7 (77.8)	17 (81.0)	
Early allograft dysfunction with infection			< 0.001
Yes	0 (0.0)	4 (19.0)	
No	9 (100.0)	17 (81.0)	

## Discussion

A comparison by multivariate analysis between the hospital mortality group and the no hospital mortality group found high donor age, high blood loss and annual surgery volume < 30 liver transplantations to be statistically significant risk factors. Annual LDLT volume was less than 10 cases in the first 5 years of our transplantation center (Fig. 1). It was a little surprising that the outcome for the period with < 20 annual cases and the outcome for the period with ≥ 20 annual cases were not statistically different. When the case volumes were small, we were more cautious in that selected recipients had lower MELD scores, the GRWR was kept above 0.8% and safe donor grafts were used (normal vessel or biliary tract anatomy). The first 15–20 LDLT cases are associated with a significant surgery learning curve [16, 17]. Annual surgery volume grew to 20–30 cases in the 6th and 7th years; there were more urgent patients with acute liver failure conditions, including some recipients with rapid development of hepatic dysfunction associated with encephalopathy or renal failure. By putting the recipient in a positive pressure isolation room with 2 beds in the intensive care unit, the nosocomial infection risk was reduced. Over the period with 30–50 annual cases, we used soft power of critical care training and continuing education of the staffs. An important point is that multidisciplinary characteristics such as integration and organization management add to the operative learning curve (multiple anastomoses of vessel or biliary tract anatomy). The training of the multidisciplinary staffs covered color Doppler techniques, anesthesia, critical care, rejection identification and infection treatment. We have a combined intensivist in infectious diseases and critical care, and we established a specialized liver transplantation ward. Thus, we had put infrastructure in place to ensure favorable outcomes. Operative time, blood loss, re-operation, intensive care unit days, length of stay and hospital mortality decreased significantly after annual surgical volume reached 30 cases. After improving soft power and care quality, our physical selection criteria became less strict in regard to senior recipients, donor age, the GRWR lower bound (range 0.55–0.8), ABO-incompatible liver transplantation, and multiple hepatic duct or portal vein anastomoses, which were not limiting factors or difficult techniques. However, neither portal vein complications nor early allograft dysfunction led to a significantly higher mortality rate. In addition, the hospital mortality rate when the annual surgical volume was less than 20 liver transplantations was 10.5%, and it decreased to 5.4% and 5.1% when annual surgical volume was ≥ 30 and ≥ 50 liver transplantations, respectively.

The complications with the highest mortality rates were cerebrovascular problems, including subarachnoid and intracerebral hemorrhages [18, 19]. Common early postoperative complications following LDLT include thrombosis in reconstructed major blood vessels (portal vein or hepatic artery reconstructed with an artificial vascular graft or cryopreserved vein grafts) [17]. In the absence of ongoing bleeding after operation, our center considered maintaining an INR between 1.5 and 2, a platelet count > 50,000/μL and a fibrinogen level > 100 mg/dL as satisfactory. Hypertension occurs usually in the initial treatment when the systolic blood pressure is greater than 160 mmHg or the diastolic blood pressure is greater than 100 mmHg. In the general population, intracranial hemorrhage may occur in association with coagulopathy, acute hypertension or chronic hypertension. An intracranial or subarachnoid hemorrhage after liver transplantation that requires immediate craniotomy and removal of a hematoma may be combined with nosocomial infections and result in high mortality. Postoperatively, blood pressure and fibrinogen levels can be monitored closely to help prevent post-transplant intracranial hemorrhages [18].

In our center’s policy, when old age, cardiomegaly, history of coronary artery disease (CAD), or massive ascites is a trait in an alcoholic cirrhosis patient, the patient undergoes regular electrocardiograms and echocardiographies for pre-operative cardiovascular assessment of LT. If the patient is an LT candidate, then dobutamine stress myocardial perfusion scanning is performed for detection of CAD. Active coronary artery disease is a relative contraindication to liver transplantation and at a minimum should be treated as aggressively as possible preoperatively (stenting, angioplasty). For high cardiopulmonary risk patients, we evaluated their hemodynamic measurements by pulmonary artery catheter. Cardiac dysfunction and moderate to severe portopulmonary hypertension (mean pulmonary arterial pressure ≥ 35 mmHg and elevated pulmonary vascular resistance) were diagnosed and were considered contraindications of liver transplantation [20]. Cardiovascular complications occurred in 8 patients after LDLT. Two patients survived. One survivor had arrhythmia with atrial fibrillation, paroxysmal supraventricular tachycardia, ventricular tachycardia and ventricular fibrillation in the first week after liver transplantation. The recurrent arrhythmia was poorly controlled by anti-arrhythmia treatment and defibrillation. Careful laboratory monitoring and supplementation were warranted; electrolytes were provided to maintain a normal level. An echocardiography showed moderate mitral regurgitation and tricuspid regurgitation. Still, anti-arrhythmia treatment did not prevent the arise of severe bradycardia with atrioventricular block. The discontinuation of antiarrhythmic drug therapy showed no significant improvement, and a cardiologist suggested inserting temporary pacemakers. The other surviving patient’s electrocardiogram presented ST elevation and increased levels of myocardial enzymes. Percutaneous coronary intervention was done to exclude an obvious problem.

The other 6 cases with cardiovascular complications resulted in hospital mortality. One case involved the rupture of an aortic mycotic aneurysm after transplantation. This patient had a diagnosis of abdominal mycotic aneurysms and infection by salmonella species in pre-transplantation image evaluation. Treatments of patients with mycotic aneurysms caused by salmonella should include antibiotic therapy and surgery [21]. This patient had a high MELD score and massive ascites; a cardiovascular surgeon recommended antibiotic therapy and endovascular stent repair after liver transplantation. On the 12th day after liver transplantation, a rupture of an aortic mycotic aneurysm resulted in emergency surgery; the cause of death was hemorrhage. Surgery as an early-stage prevention of aneurysm rupture may decrease morbidity or mortality [21]. In three cases, cardiac arrest occurred within 2–5 min after reperfusion in intraoperative status. They experienced high-quality cardiopulmonary resuscitation, but their hemodynamic conditions remained unstable. They were then placed on extra-corporeal membrane oxygenation (ECMO). The 2nd case had hyperkalemia combined with acidosis due to a massive intraoperative blood transfusion and renal failure history. In the 3rd and 4th cases, CAD and pulmonary embolism diagnoses were ruled out by percutaneous coronary intervention. Cardiac death within 5 min after graft reperfusion may result from many possible causes, including hyperkalemia, acidosis, pulmonary embolism, hypothermia, arrhythmia, cardiac tamponade, acute heart failure, and myocardial infarction [22, 23]. We finally reached the diagnoses of acute coronary syndrome. The patients’ conditions were hemodynamically stable, and their ECMOs were successfully removed after a few days. However, they suffered from cardiac arrest in the ICU. In the 5th case, we diagnosed acute coronary syndrome by percutaneous coronary intervention, and the 6th case showed mild pulmonary hypertension by echocardiography. Both cases developed septic shock. CAD was found to be the leading cause of early mortality, and it was followed by infection [24]. Cardiovascular complications are the main cause of non-graft-related mortality after LT.

Early allograft dysfunction occurred in 10 cases after LDLT. Successful liver function recovery occurred in 2 cases. Six cases resulted in additional liver transplantations (2 case deaths from severe sepsis), and 2 cases resulted in death while waiting for graft liver. An analysis revealed that causes of early allograft dysfunction are high MELD score (≥ 35) combined with low GRWR (range 0.67–0.69), hepatic steatosis (moderate) in the donor graft and senior donor age (59 and 65 years). The donor's age and moderate and severe steatosis were already established risk factors for early allograft dysfunction or primary graft dysfunction or non-function [25–29]. Donor age (≥ 50 years) was an independent risk factor to affect regeneration after transplant [25]. Effective graft regeneration may be associated with stem cells or progenitor cells in an elderly donor’s liver [9]. The best survival results in our study were observed when the MELD score was below 15. Those with low MELD scores (n = 2, MELD 7 and 13) could better tolerate an initial graft dysfunction than the recipients with medium or higher MELD scores who were not achieving successful transplantations due to hepatic steatosis (moderate), senior donor age, or a small-sized graft (GRWR < 0.7) succumbing to early allograft dysfunction or graft failure. Values above this limit constitute important factors associated with post-transplant hospital mortality [27–29]. This should also be taken into account when deciding whether to transplant.

Many studies have analyzed the association between hospital mortality and transplantation volume in centers [1, 5]. Our study analyzed the association between surgeon volume (LT cases) and hospital mortality. The early phase of a surgeon’s LDLT practice involves frustration and numerous hardships. Massive blood loss, long operative time, prolonged mechanical ventilation, and post-operative complications lead to sepsis and early hospital mortality. Our center is a non-metropolitan hospital; decisive transplant leadership and team staff centripetal force were absolutely essential. Transplant leadership made decisions on valid multidisciplinary integration and organization management. We found a statistically significant association between hospital mortality and surgical volume. This study has some limitations. This study excluded deceased donor liver transplants. Our center only performed 4–8 deceased donor liver transplants per year due to a shortage of available organs in Taiwan, and initial development was lower liver graft. An LDLT department needs multidisciplinary integration and organization management. In sharing the development experience of our center, we believe all emerging centers must have access to mentoring while developing an LDLT program.

## Conclusions

Donor age, blood loss and a surgical volume < 30 yearly liver transplantations were significant risk factors from the pre- and peri-operative periods. A comparison showed that annual surgical volumes ≥ 30 liver transplantations had significantly lower blood loss, operative time, intensive care unit days, length of stay, re-operation rate, and hospital mortality than annual surgical volumes < 30 liver transplants. During the annual surgical volume ≥ 30 period, we practiced more soft power; this may impact quality of care and therefore transplant outcomes.

## Data Availability

Ethical restrictions prohibit the authors from making the data publicly available in order to protect patient confidentiality and privacy. Interested researchers can submit data access requests to the Changhua Christian Hospital Institutional Data Access.

## Abbreviations

LT: Liver transplantation
LDLT: Living donor liver transplantation
MELD: Model for end-stage liver disease
IVC: Inferior vena cava
INR: International normalized ratio
ALT: Alanine aminotransferase
GRWR: Graft-recipient weight ratio
CAD: Coronary artery disease
ECMO: Extra-corporeal membrane oxygenation
